# Insights into Avian Incomplete Dosage Compensation: Sex-Biased Gene Expression Coevolves with Sex Chromosome Degeneration in the Common Whitethroat

**DOI:** 10.3390/genes9080373

**Published:** 2018-07-26

**Authors:** Hanna Sigeman, Suvi Ponnikas, Elin Videvall, Hongkai Zhang, Pallavi Chauhan, Sara Naurin, Bengt Hansson

**Affiliations:** Department of Biology, Lund University, Ecology Building, 223 62 Lund, Sweden; hanna.sigeman@biol.lu.se (H.S.); suvi.ponnikas@biol.lu.se (S.P.); elin.videvall@biol.lu.se (E.V.); ho6021zh-s@student.lu.se (H.Z.); pallavi.chauhan@biol.lu.se (P.C.); sara.naurin@fs.lu.se (S.N.)

**Keywords:** sex chromosomes, recombination, degeneration, gene expression, dosage compensation

## Abstract

Non-recombining sex chromosomes (Y and W) accumulate deleterious mutations and degenerate. This poses a problem for the heterogametic sex (XY males; ZW females) because a single functional gene copy often implies less gene expression and a potential imbalance of crucial expression networks. Mammals counteract this by dosage compensation, resulting in equal sex chromosome expression in males and females, whereas birds show incomplete dosage compensation with significantly lower expression in females (ZW). Here, we study the evolution of Z and W sequence divergence and sex-specific gene expression in the common whitethroat (*Sylvia communis*), a species within the Sylvioidea clade where a neo-sex chromosome has been formed by a fusion between an autosome and the ancestral sex chromosome. In line with data from other birds, females had lower expression than males at the majority of sex-linked genes. Results from the neo-sex chromosome region showed that W gametologs have diverged functionally to a higher extent than their Z counterparts, and that the female-to-male expression ratio correlated negatively with the degree of functional divergence of these gametologs. We find it most likely that sex-linked genes are being suppressed in females as a response to W chromosome degradation, rather than that these genes experience relaxed selection, and thus diverge more, by having low female expression. Overall, our data of this unique avian neo-sex chromosome system suggest that incomplete dosage compensation evolves, at least partly, through gradual accumulation of deleterious mutations at the W chromosome and declining female gene expression.

## 1. Introduction

Sex chromosomes have evolved from autosomes repeatedly in many taxa [1,2,3,4]. This chromosomal transformation is believed to start with the acquisition of a sex-determining mutation [1,5,6,7]. Thereafter, sex-specific mutations that are beneficial to the sex which the locus determines accumulate, leading to suppression of genetic recombination in this region of the chromosome [8,9,10]. Recombination suppression will prevent expression of these sex-specific alleles in the other sex and will retain linkage of these new sex-optimizing loci [5,9,11,12]. Typically, the suppression of recombination then continues to spread across the chromosome, ultimately resulting in two divergent, heteromorphic, non-recombining sex chromosomes (X-Y or Z-W), with only a small pseudoautosomal region (PAR) continuing to recombine [13,14]. The complete lack of recombination over a substantial part of one of the sex chromosomes (Y or W), which is only ever present in one copy (in XY males and ZW females, respectively), will lead to chromosome degradation due to accumulation of deleterious mutations [1,5,6]. That is why, today, the mammalian Y and the avian W chromosomes that were formed a long time ago are often small and gene poor [3,15,16,17].

Originally, in their autosomal state, each gene is present in two copies, one on each of the homologues of the chromosomal pair. When the Y or W degrades, however, genes are lost, leaving heterogametic XY males or ZW females with a reduced copy number of genes (a lower gene dose) than the homogametic XX females or ZZ males, and thereby with lower gene expression levels [2,18,19]. This change in gene dose is believed to be harmful due to the disruption of crucial gene networks [2,18,20,21,22,23,24]. It is further believed that the detrimental reduction in gene expression levels has triggered counter selection for increased expression of the single copies of the genes that remain—so called dosage compensation [1,19,25,26,27].

Dosage compensation has been extensively studied in vertebrates [2,3,16,18,19,28,29,30,31,32,33,34,35,36,37,38]. Some interesting patterns and differences between taxa have been found. In therian mammals—marsupials and placental mammals—dosage compensation is a relatively complete phenomenon observed over the whole chromosome through inactivation of one of the X chromosomes in females, which leads to a state where expression levels of X-linked genes are often similar between sexes despite the fact that males have only one X while females have two [2,18,19]. In birds, however, dosage compensation has not led to equal expression of Z-linked gene in the two sexes. Instead birds show incomplete dosage compensation, with females having consistently lower expression levels for a large proportion of the Z-linked genes [22,28,29,30,32,35,36,38,39]. This difference was originally described as a lack of dosage compensation in birds, a description which is at least partly correct as female birds compensate their gene expression from an expected 50% of the male dose to doses that range between 60–100% of that of the males depending on the gene [2,18,28,29,30,32,39]. Overall, sex-biased gene expression is much more prevalent on the avian Z chromosome than on autosomes, and regulation of partial dosage compensation is likely to take place by a gene-by-gene process that varies between tissues and time points [40,41] rather than as a chromosome-wide mechanism as in mammals.

The sex chromosomes in mammals and birds are ancient [2,3]. Birds and monotremes diverged around 310 million years ago (mya) and the ancestral avian Z/W chromosomes could date back to that age [2], although recent work suggest a more recent origin (around 140 mya) [3]. Analyses of such old, highly heteromorphic sex chromosome systems may bias our understanding of sex chromosome evolution in terms of degeneration and dosage compensation because the few genes that remain on the non-recombining sex chromosome, and thus can be studied, are likely to be a selected set of genes with unusual properties. Analyses of more recently sex-linked genetic material, such as neo-sex chromosomes, would be particularly valuable, because this may bring understanding about whether incomplete dosage compensation, which is typically observed on the avian ancestral sex chromosomes [22,28,29,30,32,35,36,38,39], evolves through gradual accumulation of deleterious mutations at the non-recombining W chromosome and declining female gene expression, or through more unsynchronised evolutionary events at the sequence and the expression levels, e.g., due to more complex combinations of adaptive and deleterious mutations at different genes.

Recently, we identified such a young neo-sex chromosome system in Sylvioidea songbirds [42,43]. In these birds, the homolog of zebra finch chromosome 4A (*Taeniopygia guttata*) has split approximately in half, leaving one half autosomal, while the other has fused with the ancestral Z and is now sex-linked [42]. This is the first described neo-sex chromosome in birds. We estimated the Sylvioidea neo-sex chromosome to be around 40 million years old as dated by the split between the Sylvioidea and other passerine lineages [42], but a recent phylogeny that moves the date of the Passerine radiation forward in time implies a young age: c. 19 million years [44]. Thus, the Sylvioidea neo-sex chromosome is young compared to the sex chromosomes in other bird lineages and to the ancestral part of the sex chromosome in the Sylvioidea themselves (c. 140 million years) [3,44].

In the present study, we evaluate the relatively recent evolutionary stages of Z and W sequence divergence and sex-specific gene expression in birds by analyzing gametologs (i.e., the Z and W copies of the same genes) on the Sylvioidea neo-sex chromosome in the common whitethroat (*Sylvia communis*). We generate Z and W sequences for 64 neo-sex chromosome genes from whole-genome resequencing data of both males and females. For comparison, we also include 39 genes from the ancestral part of the sex chromosome in the study. To test hypotheses of gene degradation and sequence divergence of young and old Z and W gametologs in response to loss of recombination, decrease in population size (Z: ¾; W: ¼) and loss of gene function, we analyse the rate of functional (protein changing) and silent (non-protein changing) mutations, and GC content (reflecting recombination [45]). We also generated RNAseq expression data in male and female common whitethroats for the same set of genes to test hypotheses of sex-biased gene expression and dosage compensation by evaluating whether sex-biased gene expression coevolves with the degradation of sex chromosomal genes.

## 2. Materials and Methods

### 2.1. Study Species and Sequencing

The common whitethroat (*Sylvia communis*) breeds in Europe and Asia and most populations migrate to tropical Africa during winter [46]. It is moderately sexually dichromatic and has a socially monogamous mating system [47]. DNA was extracted from a small blood sample of 2 males and 2 females mist-netted in southern Sweden (Skåne: 55°42′01″ N; 13°28′42″ E) in May 2015. Illumina sequencing libraries were prepared by NGI Uppsala and sequenced with Illumina HiSeqX (paired- end, 2 × 150 bp). A total of 738 million read pairs were obtained from the samples after quality check and adaptor removal using *Trimmomatic* v.0.36 [48] (options TruSeq3-PE.fa:2:3 0:10 leading:15 trailing:30 slidingwindow:4:20 minlen:90).

Total messenger RNA (mRNA) was extracted from brain tissue of 12 adult common whitethroats (6 males and 6 females) caught in the wild, using mist-nets and playback song, in southern Sweden (Skåne: 55°42′16 N, 13°25′52 E), in the end of May 2005 (all birds were caught prior to any egg laying). Brain tissue was chosen for the analyses because we were interested in gene expression in relation to both the sex chromosomes and dosage compensation (in this study) and sexual behaviours (in another study). Birds were in good condition and were sacrificed through decapitation to ameliorate suffering. For detailed information regarding sample handling and mRNA extraction, see Naurin et al. [35,39,49]. DNase treatment, rRNA reduction and RNA quality control were performed by Beijing Genomics Institute (BGI; Hong Kong). BGI also conducted complementary DNA (cDNA) synthesis, Illumina library preparation, indexing and library quality control, cluster generation and sequencing using Illumina HiSeq 2000 (single-end, 49 bp). A total of 225 million raw reads were obtained for the 12 samples.

Ethical approval was obtained from the Malmö-Lunds djurförsöksetiska nämnd (M 45-14) [35,39].

### 2.2. DNA Analyses

#### 2.2.1. Extracting Gametolog Sequences

The Illumina reads of the four common whitethroat individuals were aligned to the zebra finch genome (tgu3.2.4, downloaded from Ensembl [50]) with bwa mem v0.7.15 [51] using -M for marking shorter split reads as secondary and -R for downstream compatibility. The resequencing data aligned to the reference genome with a success rate between 88.07% to 88.66%. The proportion of properly aligned reads ranged from 81.34% to 82.02% between the samples. The bwa-aligned reads were sorted with samtools v.1.4 [52] and deduplicated with MarkDuplicates in picardtools v2.9.0 (http://broadinstitute.github.io/picard). We used standard settings for the alignment despite the evolutionary distance between the zebra finch and the common whitethroat, which is around c. 22 million years [44], as we were only interested in the exon regions which are in general well conserved. We used the gene annotation (in gff3 format) from the zebra finch genome (tgu3.2.4, downloaded from Ensembl [50]) to get genome coordinates for the exons of each transcript in the genome. Freebayes v0.9.9 [53] was used to call variants within the exon regions through providing a bed file with the genomic coordinates. The setting --report-monomorphic was used to be able to phase the whole gene sequence.

An in-house script (Supplementary Document S1) which extracts the gene sequences from the variant file (VCF format) based on sex-specific variant patterns was then used to phase the data into a Z and a W sequence. The script evaluates each site in the variant file containing the female and male variant information and works as follows: At monomorphic sites, without variation in the common whitethroat compared to the zebra finch, the reference allele from the zebra finch genome was extracted. If the quality or read depth of the variant was <20, or if the genotypes of any of the four individuals were missing, a number of *Ns* equal to the number of base pairs in the variant was extracted to avoid analyses of uncertain nucleotides. Any tri-allelic sites within the four individuals were also replaced with *Ns*. If the variant passes these filters, the Z and W allele for each variant was determined based on the male and female allele composition. We went through every possible allele combination for the four individuals and decided on the criteria for when a variant can be safely determined as a Z or W allele. Ambiguous sites were marked as *N*. For example, if both males were homozygous for the reference allele in the zebra finch genome, and both females were heterozygous with one reference allele and one variant, the Z allele was set as the reference and the W allele as the variant. Similarly, if both males were homozygous for a variant and the females were heterozygous, the Z allele was set as the variant and the W allele as the reference. To be more confident in our W sequences, we allowed no difference between the female genotypes, as this dataset only contains two W chromosomes in total. For the Z allele, of which there are six in this dataset, we extracted the most common allele. The criteria for Z and W allele extraction are provided in the Supplementary Materials (Supplementary Table S1).

To confirm that both Z and W gametologs were present in the data, we calculated the genome coverage for every exon using bedtools v.2.27.1 multicov [54]. The genome coverage values were normalized for each individual by dividing the total number of aligned reads in the sample having the highest amount of reads with the total number of reads in the other samples. A single genome coverage value for each transcript was calculated as the mean coverage between all exons belonging to that transcript. Transcripts where females had less than 70% of the male coverage were removed from further analysis, as this suggests absence of a W gametolog.

#### 2.2.2. Gametolog Sequence Alignment and Processing

TransDecoder.Longorfs in TransDecoder v3.0.1 (https://github.com/TransDecoder/TransDecoder/) was then used to get the longest open reading frame for each of these sequences (with additional evidence by using blastp against the NCBI non-redundant [55] (downloaded 12 June 2017) database (-max_target_seqs 1, -outfmt 6 -evalue 1 × 10^−5^) and hmmscan in Hmmer v.3.1b2 [56] to find similar sequences in the Pfam database (version 31.0, downloaded 11 July 2017) [57], retaining only the single best open reading frame for each sequence by using TransDecoder. Predict (setting: --retain_pfam_hits, --retain_blastp_hits, --single-best-orf)). The zebra finch cDNA transcript corresponding to each transcript was added as a third sequence. *Prank* v.150803 [58] was used to align the sequences (options -codon). Any *Ns* were replaced by gaps (-) so that any codons containing gaps would be removed with gblocks v0.91b [59] (using options -t=c -p=y). The program codeml from the PAML v4.9 package [60] was used to get maximum likelihood pairwise calculations of dN/dS between the three sequences with an estimated kappa and codon frequency F3X4. GC-content for all sequences after alignment was calculated using the EMBOSS v6.5.7 [61] program infoseq.

Transcripts were selected for further analysis under the following criteria: (i) a minimum length of 500 bp; (ii) a likelihood score (estimated by codeml) smaller than −500; and (iii) a minimum dS score of 0.01 (correct maximum likelihood dN/dS estimates rely on some sequence divergence). Furthermore, all alignments between the common whitethroat sequences and the zebra finch sequences were manually inspected and sequences with bad alignments were removed. In cases where one gene had more than one transcript, the longest transcript was selected for further analysis. In the end, 64 genes from the neo-sex chromosome remained, and 39 genes from the ancestral sex chromosome (gene names, chromosome position, etc., for these genes are given in Supplementary Table S2, the GC statistics in Supplementary Table S3 and gene ontology (GO) annotations in Supplementary Table S4). The DNA sequences of all 103 analysed genes are provided as unaligned sequences in Supplementary Document S2 (fasta format) and as aligned sequences without gaps in Supplementary Document S3 (multiple sequence alignment format). 

### 2.3. Gene Expression Analyses

The RNA sequencing reads were quality screened with FastQC v. 0.10.1 and a total of 151 million reads (out of 217 million reads in total, 69.52%) were mapped to the zebra finch genome (tgu3.2.4, downloaded from Ensembl [50]) using TopHat2 v.2.0.9 [62] with parameters allowing for 20% mismatch rate in order to account for the divergence between the species. The annotated zebra finch gene model (based on Ensembl gene IDs) was used as a guide in TopHat2 [62] (parameter—transcriptome-index). Mapped reads were sorted using samtools [52] and counted using HTSeq v. 0.5.3p9 [63] with default parameters. Expression levels of all genes were normalized per individual according to the DESeq2 manual v. 1.0.19 [64] to account for library size differences. We used the recommended negative binomial Wald test in DESeq2 to test for differential expression between males and females. The *p*-values of all genes were corrected with the Benjamini and Hochberg false discovery rate, and the genes with a corrected *p*-value < 0.05 were regarded as significantly differentially expressed. After removal of transcripts without adjusted *p*-values, 12911 transcripts remained (out of 18618 transcripts in total from the zebra finch annotation).

### 2.4. Statistics

Statistical analyses were performed in R v.3.4.4 [65]. We used non-parametric tests (Wilcoxon signed-rank tests, Wilcoxon rank-sum tests and Spearman’s rank correlations) when data were not normally distributed.

## 3. Results

### 3.1. Sequence Divergence

The divergence (dS, dN and dN/dS) between the Z and W for each gametolog on the neo-sex chromosome (*n* = 64) and the ancestral sex chromosome (*n* = 39) in the common whitethroat are shown in Supplementary Table S2. For gametologs on the neo-sex chromosome, the rate of non-synonymous mutations (dN) (median = 0.009) was significantly lower on average than the rate of synonymous mutations (dS) (median = 0.055; Wilcoxon signed-rank test, *p* < 0.001). This was also true for gametologs on the ancestral sex chromosomes (dN: median = 0.019; dS: median = 0.189; *p* < 0.001). There was a strong correlation between dN and dS on the neo-sex chromosome (Spearman’s rank correlation: *r*_S_ = 0.41, *n* = 64, *p* < 0.001), but not for the ancestral sex chromosome (*r*_S_ = 0.23, *n* = 39, *p* = 0.15). The neo-sex gametologs were less diverged (based on dS) on average than the gametologs on the ancestral sex chromosome (comparing median dS between the neo-sex chromosome and ancestral sex chromosome; Wilcoxon rank-sum test: *p* < 0.001; Supplementary Table S2).

**Figure 1:**
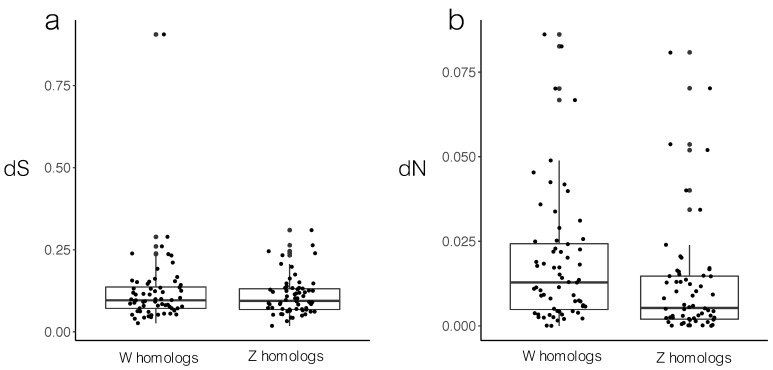
(**a**) Rate of synonymous substitutions (dS) and (**b**) non-synonymous substitutions (dN) between homologous genes in the zebra finch and the common whitethroat, i.e., zebra finch chromosome 4A genes and common whitethroat neo-W and neo-Z sequences. There was no statistical difference for dS (*p* = 0.24), whereas dN differed between W and Z (*p* < 0.001).

To understand if the sequence divergence between the common whitethroat neo-sex chromosome is driven by the W chromosome or the Z chromosome, we compared the dN and dS between these genes and the zebra finch homologs. Since the neo-sex chromosome formed (c. 19 mya) after the split with the zebra finch (c. 22 mya), the Z and W sequence in the common whitethroat should be equally distantly related to the zebra finch homologs in the absence of selection. We found that while the common whitethroat W and Z sequences had equal synonymous substitution rate (dS) to the zebra finch sequences (paired *t*-test: *t* = 1.18, *p* = 0.24; Figure 1a), the common whitethroat W sequences had higher non-synonymous substitution rate (dN) than the Z sequences (paired *t*-test: *t* = 6.87, *p* < 0.001; Figure 1b). This implies that the neo-W sequences have diverged more than the neo-Z sequence from a functional point of view. A similar analysis was not possible for the ancestral gametologs since the ancestral sex chromosome formed much earlier (c. 140 mya) than the split between common whitethroat and zebra finch (c. 22 mya), and since there are no W genes in the zebra finch annotation.

**Figure 2:**
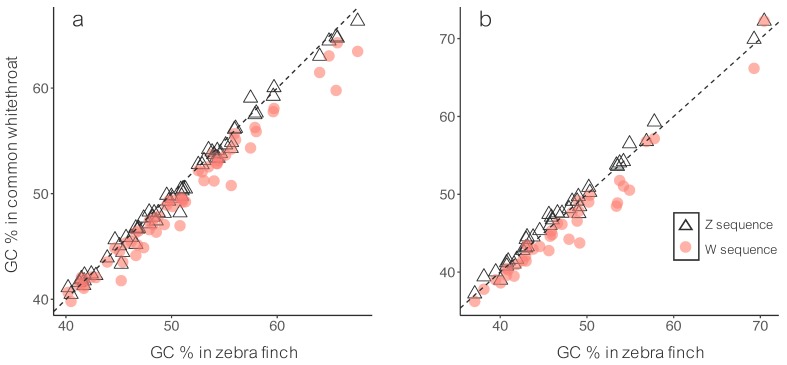
GC content (%) for common whitethroat gametologs plotted against the GC content for the zebra finch homologs. The black triangles are common whitethroat Z sequences and red circles are common whitethroat W sequences. (**a**) Neo-sex chromosome genes, corresponding to zebra finch chromosome 4A, and (**b**) ancestral sex chromosome genes, corresponding to zebra finch chromosome Z.

The W sequences (median = 49.05) had significantly lower GC than the Z sequences (median = 49.67) on the neo-sex chromosome (Wilcoxon signed-rank test, *p* < 0.001; Figure 2a). This was also true for the ancestral sex chromosome (W: median = 44.61; Z: median = 47.42; Wilcoxon signed-rank test, *p* < 0.001; Figure 2b). We also compared these GC-values to corresponding sequences in the zebra finch, i.e., sequences on zebra finch chromosome 4A for the neo-sex chromosome and sequences on zebra finch chromosome Z for the ancestral chromosome. The GC-content for zebra finch chromosome 4A (median = 50.03) was higher than both the neo-sex W (Wilcoxon signed-rank test, *p* < 0.001) and the neo-sex Z (*p* = 0.003) in the common whitethroat. The GC-content for zebra finch chromosome Z (median = 46.00) was higher than the ancestral W (Wilcoxon signed-rank test, *p* < 0.001) but lower than ancestral Z (*p* < 0.001) in the common whitethroat.

**Figure 3:**
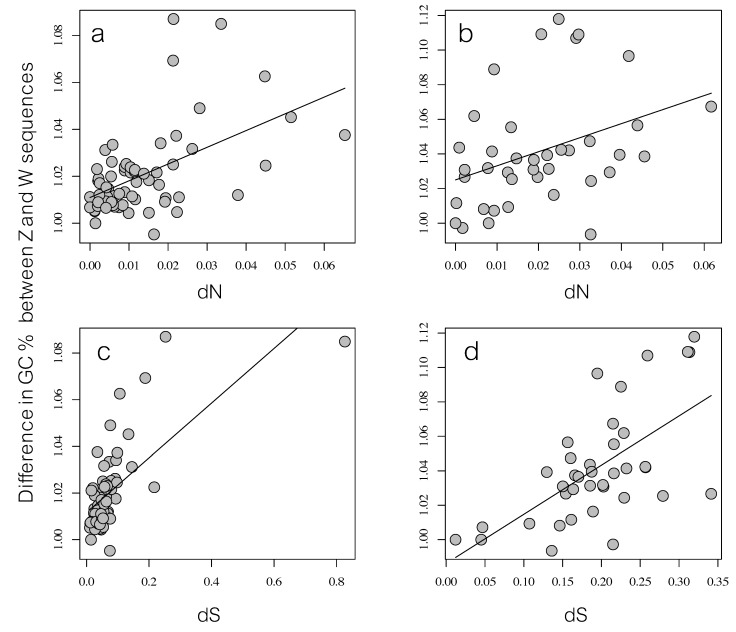
The difference in GC-content between the Z sequence and W sequence (Y axis) plotted against the sequence divergence (X axis) of common whitethroat gametologs. The panels show the Z-to-W GC-content and degree of functional divergence (dN) for (**a**) the neo-sex chromosome and (**b**) the ancestral sex chromosome, and non-functional (dS) divergence for (**c**) the neo-sex chromosome and (**d**) the ancestral sex chromosome.

Furthermore, we found that the difference in GC-content (calculated as the GC% for the Z sequence divided by the GC% for the W sequence) increased with increasing sequence divergence between the Z and W gametologs on the neo-sex chromosome (i.e., neo-Z and neo-W) in the common whitethroat: this was true for both dN (*r*_S_ = 0.47, *p* < 0.001; Figure 3a) and dS (*r*_S_ = 0.62, *p* < 0.001; Figure 3c). The same correlations were significant for the ancestral sex chromosome (dN: *r*_S_ = 0.41, *p* = 0.010; dS *r*_S_ = 0.57, *p* < 0.001; Figure 3b,d).

### 3.2. Gene Expression

**Figure 4:**
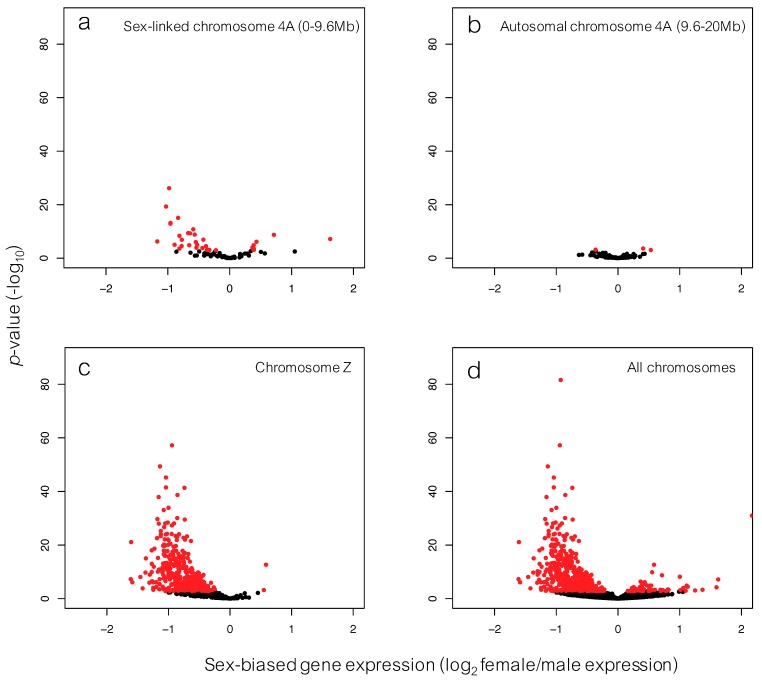
Volcano plot showing log_2_ female/male gene expression (values > 0: female-biased genes; values < 0 male-biased genes) and significance levels. Red colour marks significant genes (p_adj_ < 0.05). The genes located on chromosome 4A were divided into two parts: (**a**) those on the first 9.6 Mb of chromosome 4A, i.e., the neo-sex chromosome, and (**b**) those on the autosomal part of chromosome 4A. Also, shown are (**c**) genes located on chromosome Z, and (**d**) genes over the whole genome.

Of 12,911 genes in the data set, 470 (3.64%) were significantly differentially expressed between males and females (Figure 4; Supplementary Table S5). Of the 470 significantly differently expressed genes, 402 had higher expression in males and 68 had higher expression in females. The majority of these 470 genes were located on the zebra finch sex chromosome: 339 were located on the Z chromosome and 12 were located on Z chromosome scaffolds that are not confidently placed in the genome version. The chromosome with the second highest number of differentially expressed genes, 39 genes, was chromosome 4A (38 genes on chromosome 4A and 1 on a 4A-linked but unplaced scaffold). Of these genes, 35 were located within the first 9.6 Mb of chromosome 4A, i.e., the neo-sex chromosome region.

The mean expression on the neo-sex chromosome 4A (i.e., the first 9.6 Mb) was lower in females than in males (mean ± SE log2 female/male gene expression: −0.15 ± 0.04, *n* = 112). The gene expression was also lower in females than in males on the ancestral sex chromosome (mean ± SE log_2_ female/male gene expression: −0.59 ± 0.02, *n* = 597). The difference in gene expression between the sexes was statistically significant for both the neo-sex chromosome (paired *t*-test of female and male gene expression: *t* = −2.38, *n* = 112, *p* = 0.019), and the ancestral sex chromosome (paired *t*-test of female and male gene expression: *t* = −14.06, *n* = 597, *p* < 0.001). The ancestral sex chromosome had on average significantly more pronounced sex-biased gene expression (i.e., less expression in females than in males) than the neo-sex chromosome (Welch two sample *t*-test: *p* < 0.001; ancestral sex chromosome: *n* = 597 genes; neo-sex chromosome: *n* = 112 genes).

Of the genes that were analysed for sequence divergence (neo-sex: *n* = 64; Z: *n* = 39), the mean expression in females was slightly lower than in males on the neo-sex chromosome (mean ± SE log2 female/male expression: −0.02 ± 0.05, *n* = 64) and significantly lower in females for the ancestral sex chromosome (mean ± SE log2 female/male expression: −0.20 ± 0.05, *n* = 39). At the individual gene level, 16 genes had significantly different expression between males and females on the neo-sex chromosome and 14 on the ancestral Z chromosome. The difference in gene expression between the sexes was statistically significant for the ancestral sex chromosome (paired *t*-test: *t* = −2.18, *n* = 39, *p* = 0.036), but not for the neo-sex chromosome (paired *t*-test: *t* = −0.03, *n* = 64, *p* = 0.98).

**Figure 5:**
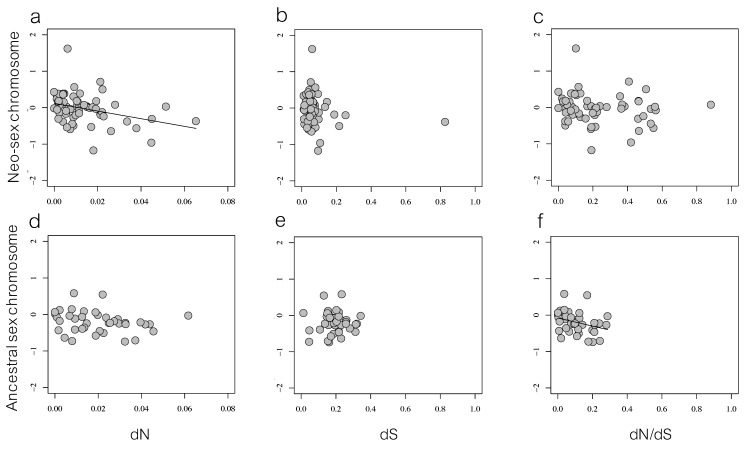
Association between female-to-male gene expression ratio and degree of sequence divergence (dN, dS and dN/dS) for common whitethroat gametologs on the neo-sex chromosome (**a**–**c**) and on the ancestral sex chromosome (**d**–**f**). Lines indicate significant correlations ((**a**) *p* = 0.009; (**f**) *p* = 0.013).

The degree of sex-biased expression (log_2_ female/male expression) of neo-sex chromosome genes was significantly negatively correlated with the degree of functional divergence between the gametologs, dN (*r*_S_ = −0.33, *n* = 64, *p* = 0.009; Figure 5a). There was no significant correlation between sex-biased gene expression and degree of non-functional divergence, dS (*r*_S_ = −0.21, *n* = 64, *p* = 0.097; Figure 5b), or between dN/dS and sex-biased gene expression (*r*_S_ = −0.21, *p* = 0.097; Figure 5c), for genes on the neo-sex chromosome. On the ancestral sex chromosome there were no correlations between differential gene expression between males and females and dN or dS between gametologs (dN: *r*_S_ = −0.31, *n* = 39, *p* = 0.054; dS: *r*_S_ = −0.01, *n* = 39, *p* = 0.97), but there was a significant correlation between dN/dS and sex-biased gene expression (dN/dS: *r*_S_ = −0.40, *n* = 39, *p* = 0.013; Figure 5d–f).

## 4. Discussion

### 4.1. Sequence Divergence

After recombination ceases, the sex chromosomes evolve independently and are expected to diverge. This pattern is evident in our study of the relatively recent evolutionary stages of Z and W sequence divergence in the common whitethroat. We show that most Z–W gametologs on the Sylvioidea neo-sex chromosome, which was formed by a fusion event between an autosome and the ancestral sex chromosome [42] approximately 19 mya (cf. [44]), have diverged substantially from each other (dS: 0.01–0.83; Supplementary Table S2) and from another passerine species (the zebra finch; Figure 1). Not surprisingly, because recombination stopped a long time ago (c. 140 mya) [3,15], also the ancestral gametologs have diverged substantially in the common whitethroat. As expected from the lack of recombination, W showed the lowest GC levels on both the neo-sex and the ancestral sex chromosome (Figure 2). Also, the difference in GC content between the gametologs correlated with their degree of divergence (Figure 3). Recombination is known to affect the base composition through GC-biased gene conversion and positive correlations between local recombination rate and GC content is observed across many taxa, including in birds [17,45,66,67].

Sex chromosome gametolog divergence is believed to be strongly influenced by the low effective population size of the non-recombining W chromosome (¼ of the autosomal population size), which drastically increases the probability that deleterious mutations will become fixed due to genetic drift. Purifying selection can, however, delay this process and maintain functional haplotypes in the population. When comparing Z and W gametologs in common whitethroat, we found that mutations altering protein function (i.e., the non-synonymous substitution rate; dN), were much less frequent than non-protein changing (i.e., the synonymous substitution rate; dS). In fact, the majority of neo-sex chromosome genes had dN/dS ratios <0.2 and only two genes, ENSTGUG00000018438 (*ZBTB33*) and ENSTGUG00000003312 (“*novel gene*”), had values close to one or above. As expected, the neo-sex chromosome gametologs were overall less diverged (dS = 0.05) than the much older gametologs on the ancestral part of the sex chromosome (dS = 0.19) in the common whitethroat. This result was evident despite the fact that our method to select Z and W sequences by mapping Illumina reads to a reference genome could have excluded highly degenerated genes from the analysis since they are less likely to generate high quality sequences that map well to the reference genome.

The overall low dN/dS ratio of our genes strongly suggests that W gametologs are not free of selective constraint; most probably purifying selection for maintained gene function purges haplotypes with deleterious mutations from the population. However, we cannot exclude that other processes could also have played a role, e.g., gene conversion by which functional elements (exons or parts of exons) have been copied from Z to W. Directional selection for improved male function on Z gametologs would lead to increased dN and thus increased dN/dS. The generally low dN that we observe among our genes suggests that this type of selection regime is weak. However, these few gametologs in our data set that had relatively high dN and dN/dS (see Supplementary Table S2) could have been affected by directional selection on Z for improved male function, although another explanation could be that dN has increased in these genes by loss of function and relaxed selection on W.

The divergence patterns of homologous genes of the common whitethroat and an outgroup could be informative of whether selection has been acting on the Z or the W gametolog. Our results show that the common whitethroat neo-W and neo-Z sequences had similar synonymous substitution rate (dS) to the zebra finch sequences on chromosome 4A (Figure 1a). This was expected as the neo-sex gametologs that we see today have had a common history of divergence towards the homologous autosomal zebra finch genes during the period after their ancestors split (c. 22 mya) and before these sex chromosomes stopped recombining in Sylvioidea (<c. 19 mya) [42,44]. However, the common whitethroat neo-W sequences had higher non-synonymous substitution rate (dN) when compared to the zebra finch homologs, than had the neo-Z sequences (Figure 1b). This implies that the neo-W sequences have diverged more than the neo-Z sequence from a functional point of view, which supports that it is mainly the neo-W that has diverged functionally rather than the neo-Z or both the neo-Z and neo-W. A similar analysis was not possible for the ancestral gametologs since the ancestral sex chromosome formed much earlier (c. 140 mya) than the split between common whitethroat and zebra finch (c. 22 mya; cf. [44]), and since the homologous zebra finch W genes are not available (i.e., we could not calculate divergence between ancestral W in common whitethroat and the zebra finch).

### 4.2. Gene Expression

Studies of gene expression on the ancestral Z chromosome in several bird species have shown that females have significantly lower Z-linked gene expression levels than males [15,29,30,32,35,36,39,40]. This pattern of incomplete dosage compensation in birds contrasts the situation in mammals that show chromosome-wide dosage compensation so that males and females have similar levels of X-linked gene expression [19]. Since birds lack a chromosome-wide dosage compensation mechanism similar to that found in mammals, avian sex-linked gene expression is believed to evolve on a gene-by-gene basis [40,41].

We found that the majority of genes with sex-biased expression (*n* = 470) were located on the ancestral sex chromosome (*n* = 339) and on the neo-sex chromosome (*n* = 35). Most of these genes were less expressed in female than in male common whitethroats (Figure 4; Supplementary Table S5). That the genes on the ancestral sex chromosome were significantly less expressed in females compared to males supports the findings in our previous study of the same individuals where gene expression was analysed with microarrays [39]. If the genes on the neo-sex chromosome have evolved in a similar fashion as the ancestral avian sex chromosome, we would expect to again see this pattern of lower female gene expression in the neo-sex genes. Indeed, several male-biased genes were located on the neo-sex chromosome, but among the genes that we analysed for gametolog divergence females had only slightly lower gene expression on average than males. We have analysed gene expression in the brain of adult common whitethroats, and it is possible that the degree of sex-biased gene expression would have been somewhat different if another tissue would have been analysed, such as gonads (cf. [18]).

Next, we were interested in understanding whether gametolog divergence had been accompanied by sex-biased gene expression. We expected to see a lower expression in females at highly diverged genes because a loss of function in the W copy would result in the expression of a single gene copy and thus less total gene expression. In line with this expectation, there was a significant negative correlation between the female-to-male gene expression ratio and the rate of protein changing mutations (dN) at neo-sex chromosome (*r*_S_ = −0.33, *p* = 0.009), and for the ancestral sex chromosome a similar negative association between the female-to-male gene expression ratio and dN/dS (*r*_S_ = −0.40, *p* = 0.013; Figure 5).

The negative correlation between the female-to-male gene expression ratio and dN of neo-sex chromosome genes suggests that sex-linked genes are either being less suppressed as they degenerate in that female common whitethroats, or that low female expression relaxes selection so that the W gametolog can diverge more functionally. We believe that the former scenario (i.e., that females lower the expression as the W gametolog degenerates) is more likely, because it is difficult to imagine why the females would modify their gene expression of genes that has not yet diverged functionally. A similar reasoning seems true also for the ancestral sex chromosome, where we detected a negative correlation between the female-to-male gene expression ratio and dN/dS, but importantly for these genes we could not confirm that it is actually W that has diverged, rather than Z, because (as explained above) homologous zebra finch W genes are not available to study.

Our results suggest that incomplete dosage compensation, which is typically observed in avian sex chromosomes, has evolved through gradual accumulation of deleterious mutations and declining female gene expression. Both on the ancestral and the neo-sex chromosome regions, female gene expression was higher than 50% of the male expression (50% would be expected in the complete absence of dosage compensation; [2,18]). Thus, despite incomplete dosage compensation and overall low gene expression in females, it is possible that female birds achieve sufficient compensation to avoid severe disruption of gene expression networks. It is also possible that the difference in sex-linked gene expression patterns between the mammalian and avian lineages might not be driven solely by mechanistic processes related to dosage compensation, but by other processes such as differences in the level of accumulated sexual antagonism and the level of sexual selection on males (shaping different expression optima of specific sex-linked genes in females) [27,68]. Sexually antagonistic mutations beneficial to the homogametic sex (ZZ or XX) are expected to accumulate on the Z and X chromosomes [5,8,69]. However, the predominant selection of Z chromosomes in males (selected 2/3 of the time in males) will lead to a higher rate of accumulation of sexual antagonism than on X chromosomes (selected 1/3 of the time in males) because males have a higher mutation rate (due to more cell cycles in the germline) [70,71] and experience stronger sexual selection [68,72,73]. Hence, it is possible that the consistent pattern of male-biased gene expression on avian Z chromosomes might, to some extent, be due to a female response to selection for low doses of male-adapted genes rather than a lack of response to selection for dosage compensation. Some of our genes are expected, based on GO annotations, to have sex specific functions: e.g., *DIAPH2* (ENSTGUG00000002329, GO:0007292) is involved in female gamete generation; *DACH2* (ENSTGUG00000002423, GO:0046545) in development of primary female sexual characteristics; and *AR* (ENSTGUG00000002760, GO:0008584) in male gonad development (Supplementary Table S4). It has previously been shown in chicken that the degree of male-biased gene expression on the ancestral avian Z chromosome increases with time since recombination ceased [15], a result that could be in line with such a hypothesis. Since our data suggest that it is mainly the W gametologs that have diverged functionally, and thus do not support at least very strong male-adaptation of Z, we find it more likely that the pattern of incomplete dosage compensation that we observe in the common whitethroat is driven by lack of selection for complete dosage compensation.

## 5. Conclusions

By analysing divergence of gametologs and sex-biased gene expression of neo-sex chromosome genes in the common whitethroat, we have found that W has diverged functionally to a higher extent than Z, and the female-to-male expression ratio correlates negatively with the degree of functional divergence. We find it most likely that sex-linked genes are being suppressed in female common whitethroats as a response to W chromosome degradation, rather than that these genes experience relaxed selection pressure, and thus diverge more, by having low female expression. Overall our data of this unique avian neo-sex chromosome system suggest that incomplete dosage compensation, which is typically observed on avian sex chromosomes, evolves, at least partly, through gradual accumulation of deleterious mutations and declining female gene expression.

## Supplementary Materials

The following are available online at http://www.mdpi.com/2073-4425/9/8/373/s1: Supplementary Document S1: In-house bash scripts for gametolog extraction; Supplementary Document S2: Unaligned DNA sequences of analysed genes (fasta format); Supplementary Document S3: Multiple sequence alignments (MSA format) of the analysed genes; Supplementary Table S1: Criteria for gametolog extraction based on allele composition in studied female and male samples (used in Supplementary Document S1); Supplementary Table S2: Information of the genes on the neo-sex chromosome (*n* = 64) and the ancestral sex chromosome (*n* = 39) analysed in the present paper; Supplementary Table S3; GC-statistics based on the gene sequences from Supplementary Document S3; Supplementary Table S4: GO interprodomain annotations for the genes analysed in the present paper (see Supplementary Table S1 for more details); Supplementary Table S5: Number of significantly sex-biased genes on each chromosome.
